# Supplementary material for: Receptor-substrate competition for the TonB homolog FusB suggests a model for ferredoxin import
Source: J Biol Chem. 2026 May 6;302(6):113121. doi: 10.1016/j.jbc.2026.113121 (PMC13253070; doi:10.1016/j.jbc.2026.113121)
Supplement: Supporting Information [file mmc1.pdf]

Receptor-substrate competition for the TonB homologue FusB suggests a model for ferredoxin import

Marta Wojnowska, Victor Flores, Tamas Yelland, Stuart R. Fisher, Agnieszka Bogucka, Katherine Stott, Daniel Walker

Supporting Information

List of Contents

S-2. Table S1. PISA analysis of FusB complexes.

S-3. Figure S1. Sequence logos of different TonB types.

S-4. Figure S2. AlphaFold3 prediction of FusB-CTD.

S-5. Figure S3. Size exclusion chromatograms of FusB-CTD constructs.

S-6. Table S2. List of all ITC experiment types and calculated thermodynamic parameters.

S-7. Figure S4. SEC-MALS data for FusB-CTD D322N in complex with SpFer.

S-8. Figure S5. Titration of $FusA_{NTR}$-GFP into FusB-CTD variants

S-9. Figure S6. Size exclusion chromatograms showing the level of complexation of FusB-CTD with $FusA_{NTR}$-GFP.

S-10. Figure S7. Titration of SpFer into $FusA_{NTR}$-GFP.

S-11. Table S3. List of plasmids used in this study.

S-12. Table S4. List of primers used in this study.

S-13. Figure S8. Binding of different SpFer constructs to FusB-CTD.

S-14. Table S5. List of all strains from 13 different genera used in the conservation analysis. (caption only)

S-14. Table S6. TonB sequences used for the generation of sequence logos. (caption only)

S-15. Figure S9. AlphaFold3 prediction of FusA-substrate complexes.

S-16. Figure S10. Raw ITC data.

Table S1. PISA analysis of complexes. Results from PISA analysis for FusB-CTD homodimer and the complex of FusB-CTD with ferredoxin. The buried surface area and the number of residues involved is shown for individual chains, A and B. Numbers in brackets show percentage of the total surface/residues. ΔG^{diss} describes complex stability, however it could not be calculated for the heterodimer complex, possibly because the respective interface is largely composed of polar interactions and complex water networks, rather than hydrophobic contacts.

	FusB-CTD homodimer (7ZC8)	FusB-CTD-ferredoxin complex (9HI3) A = ferredoxin, B = FusB-CTD
**Buried surface area (Å^2)**	**1195**	**722**
(A) Buried surface area (Å^2)	1202 (19.5%)	728 (14.7%)
(B) Buried surface area (Å^2)	1189 (19.5%)	716 (13%)
**ΔG^{diss} (kcal/mol)**	**5.3**	**(-)**
(A) number of residues	119 (17.7%)	23 (23.7%)
(B) number of residues	120 (17.4%)	18 (20.2%)

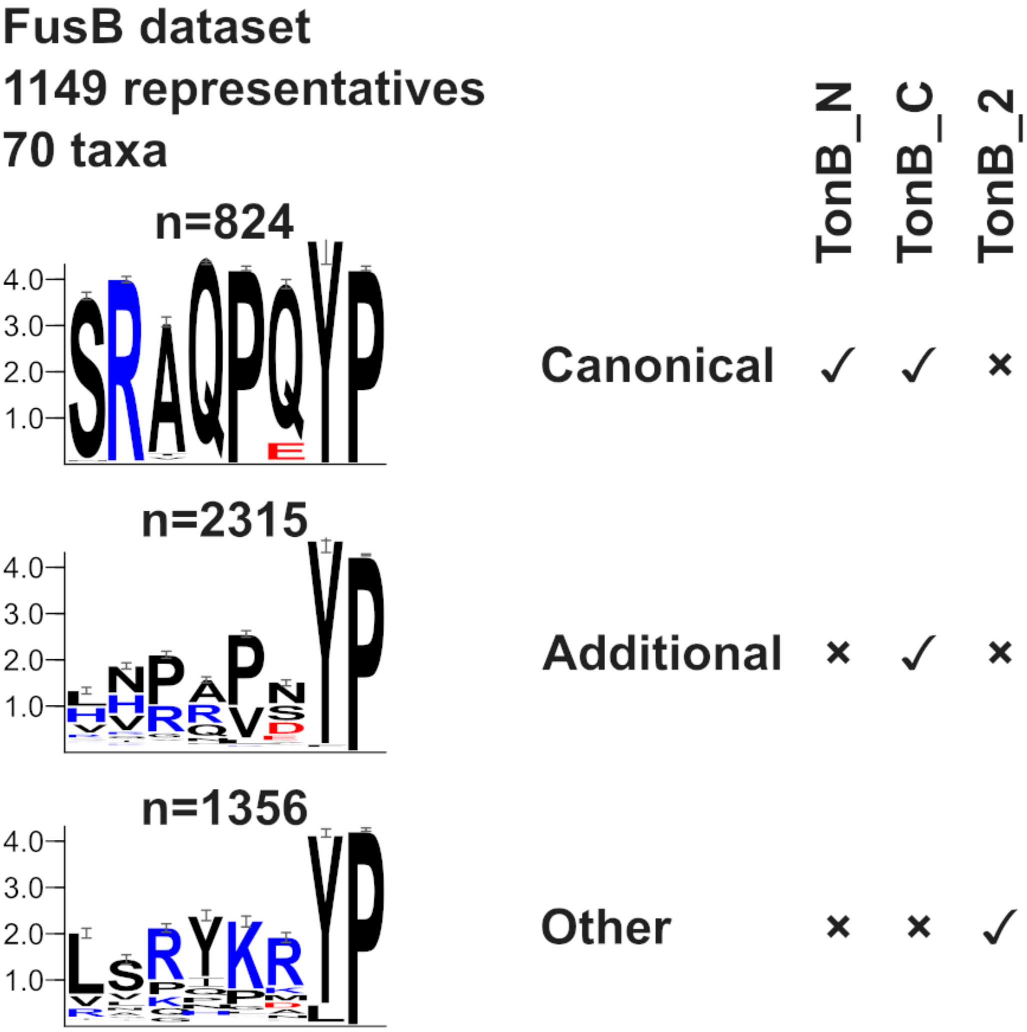

Figure S1. Sequence logos of different TonB types. TonBs from the list of 4275 sequences (Supplementary Table 6, available as a separate Excel file) were categorised as canonical, additional or other. The 7 amino acids including the conserved YP motif of each type were used to generate sequence logos. The composition (Pfam domains) of the three designated types of TonB proteins is shown on the right.

Figure S2. AlphaFold3 prediction of FusB-CTD. A –PAE (predicted alignment error) plot. B – FusB monomer prediction coloured by pLDDT (predicted local distance difference test) score. C – side by side comparison of the experimental and predicted FusB structures; R241 and D322 are shown as sticks.

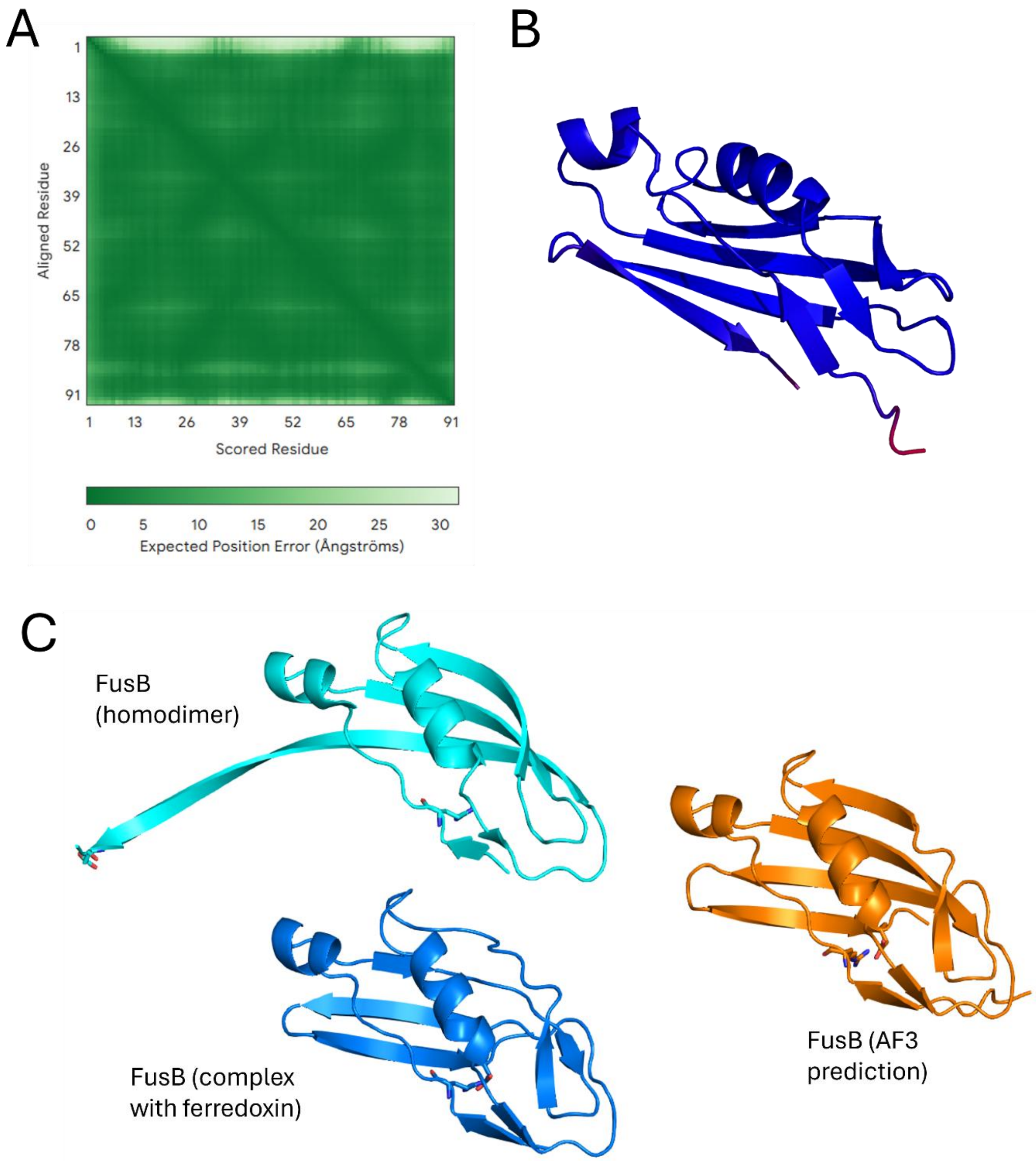

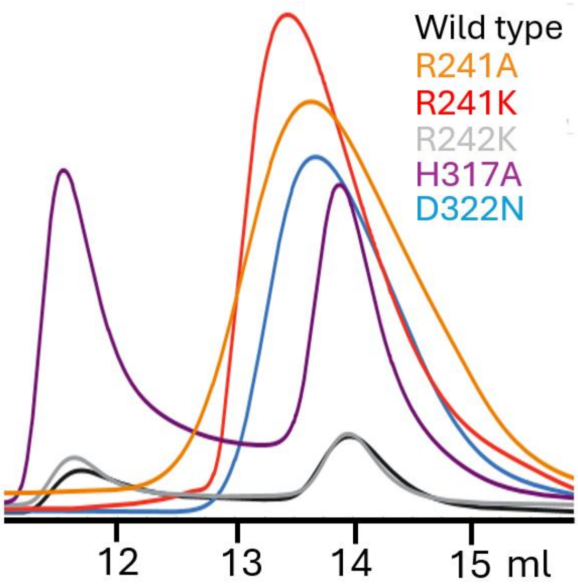

Figure S3. **Size exclusion chromatograms of FusB-CTD constructs**. Wild type (black line), R242K (grey), H317A (purple), R241K (red), R241A (orange), D322N (blue). 0.5 mg (wild type, R242K) or ~5mg protein (H317A, R241K, R241A, D322N) was loaded.

Table S2. List of all ITC experiment types and calculated thermodynamic parameters. Enthalpy contributions are all negative values. Asterisk denotes titrations that produced difficult to fit/interpret thermograms (very low signal, hyperbolic thermogram or simultaneous association/dissociation events).

Ligand (syringe)	Macromolecule (cell)	No. of titrations	K_D ±SEM (μM)	ΔH (cal mol^{-1})	ΔS (cal mol^{-1} deg^{-1})
SpFer	FusB-CTD	7	7.8±0.5	4495	8.3
SpFer-His6	FusB-CTD	2	18.1±4.6	3870	8.8
SpFer	FusB-CTD D322N	5	7.4±0.8	4320	9.1
SpFer	FusB-CTD R241K	2	25.5±3.5	3016	11
SpFer	FusB-CTD R242K	2	(-)		
SpFer F64Y	FusB-CTD	2	30.7±8.2	935	17.6
SpFer D66N	FusB-CTD	2	(-)		
SpFer E92K	FusB-CTD	2	(-)		
SpFer E(92,93)A	FusB-CTD	2	31.3±9.7	1372	16.1
SpFer	FusB-CTD H317A	2	(-)		
$FusA_{NTR}$-GFP	FusB-CTD	4	31.2±4.7*	3083	10.4
$FusA_{NTR}$-GFP	FusB-CTD D322N	3	9.4±0.6	7528	-2.2
$FusA_{NTR}$-GFP	FusB-CTD R241A	2	38.8±1.5	1511	15.1
$FusA_{NTR}$-GFP	FusB-CTD R241K	2	25.3±4.1	1940	14.6
$FusA_{NTR}$-GFP	FusB-CTD R242K	1	33	2992	11.1
$FusA_{NTR}$-GFP	FusB-CTD + SpFer	3	(-)		
$FusA_{NTR}$-GFP	FusB-CTD D322N + SpFer	2	(-)		
$FusA_{NTR}$-GFP	SpFer	1	(-)		
$FusA_{NTR}$-GFP D53A	FusB-CTD	2	31.6±6*	923	17.6
$FusA_{NTR}$-GFP D53A	FusB-CTD D322N	2	31.8±4	3069	10.3
SpFer	FusB-CTD + $FusA_{NTR}$-GFP	2	22.3±1.5*	2296	12.5
SpFer	FusB-CTD D322N + $FusA_{NTR}$-GFP	2	(-)		
SpFer	FusB-CTD + $FusA_{NTR}$-GFP D53A	2	17.7±3.2*	2416	12.5

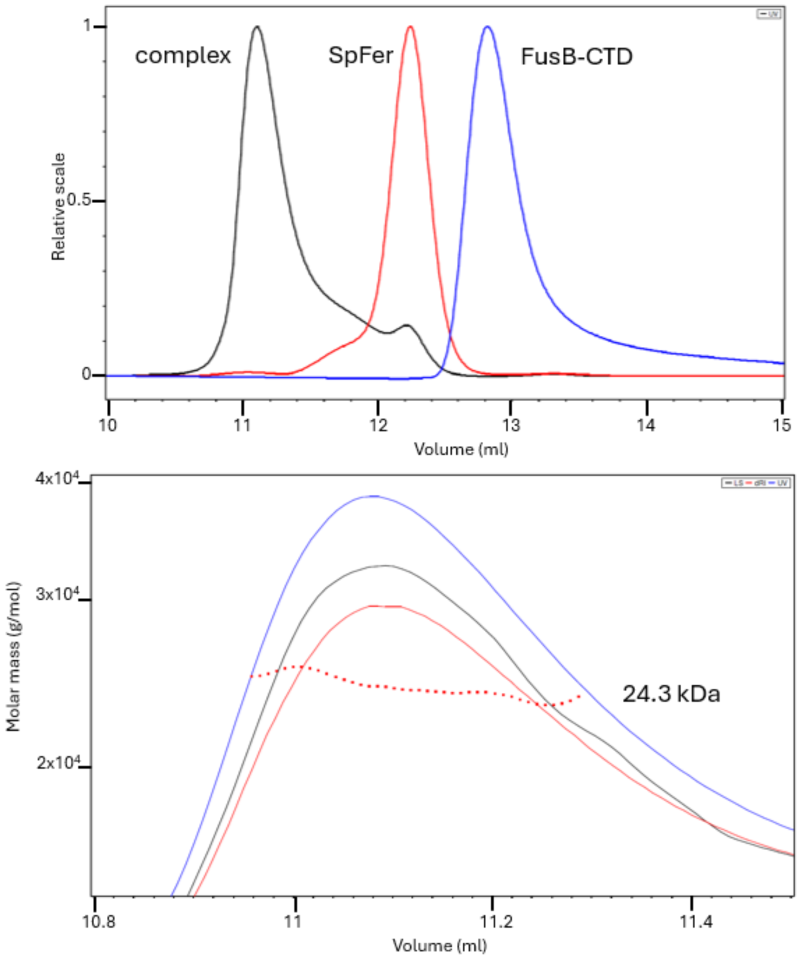

Figure S4. **SEC-MALS data for FusB-CTD D322N in complex with SpFer**. FusB-CTD variant was used to simplify the chromatogram. Top: overlay of UV chromatograms. Bottom: complex peak with molar mass (dotted line). Blue curve – UV absorption, red – refractive index, black – light scattering.

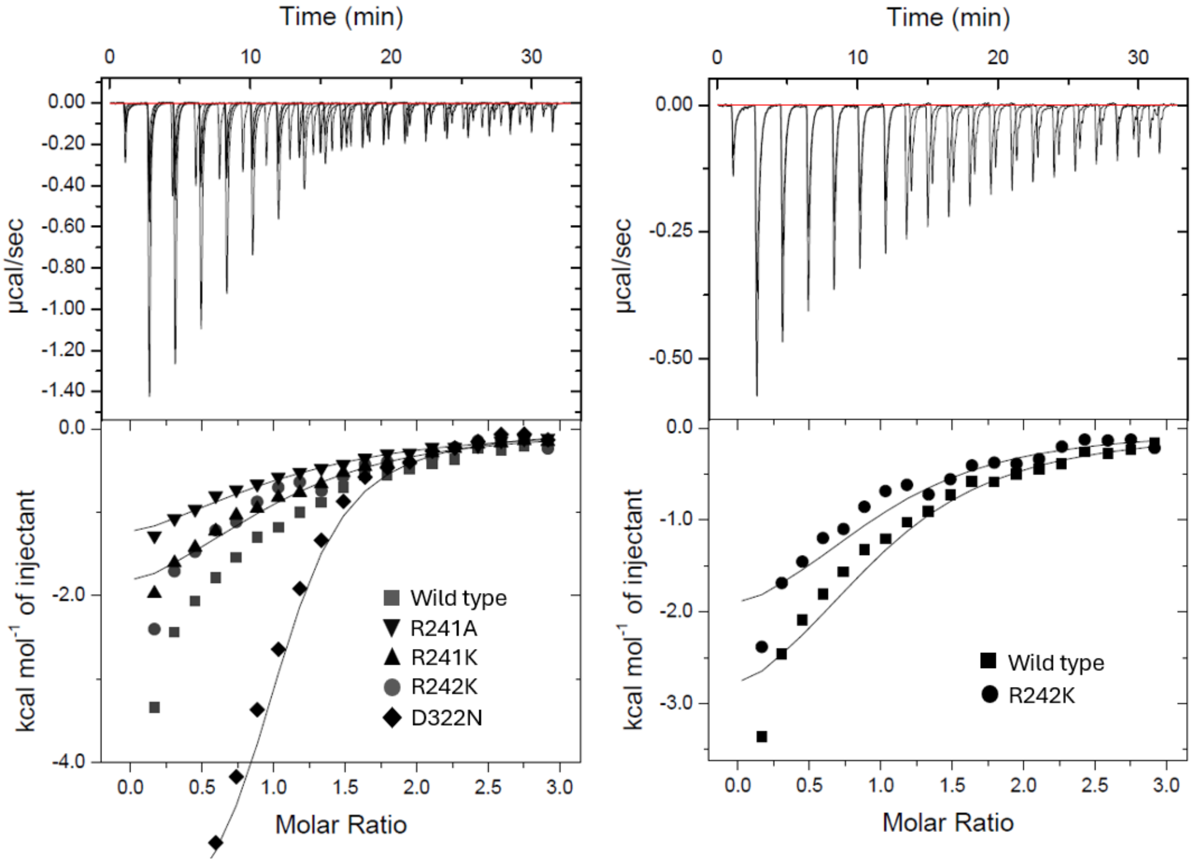

Figure S5. Titration of FusA$_{NTR}$-GFP into FusB-CTD variants. Wild type – squares, D322N - diamonds, R242K - circles, R241A - inverted triangles and R241K - triangles. The thermogram for D322N titration is also shown in Figure 4, here it is truncated to better visualise the other thermograms. Note that in the case of wild type and R242K (dark grey on the left panel) the resulting thermograms were hyperbolic in shape, and fitting resulted in stoichiometry parameter N deviating below 0.2. The right panel shows the non-optimal fits for wild type and R242K titrations with N fixed at 1.

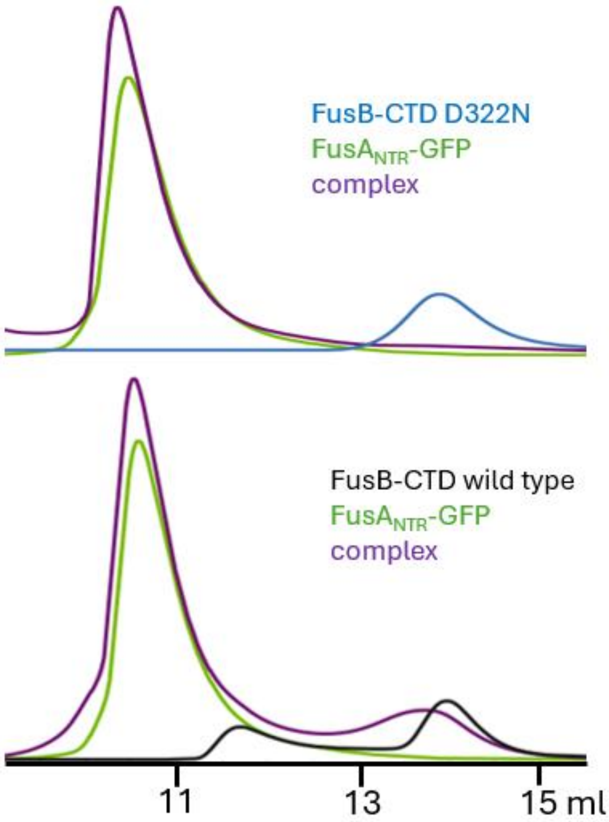

Figure S6. **Size exclusion chromatograms showing the level of complexation of FusB-CTD with FusA$_{NTR}$-GFP.** Wild type FusB – black, D322N – blue, FusA$_{NTR}$-GFP - green. In the presence of FusA$_{NTR}$-GFP the peak corresponding to D322N variant disappears completely, whereas wild type peak largely remains, indicating only partial complexation.

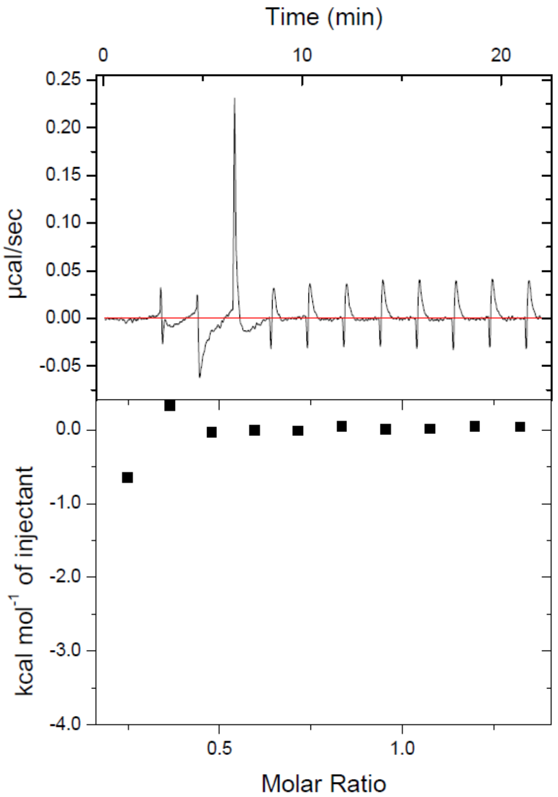

Figure S7. Titration of SpFer into $\mathbf{FusA_{NTR}}$-GFP.

Table S3. **List of plasmids used in this study.**

PLASMID	SOURCE	PARENT PLASMID
pJ404FusBCTD	Wojnowska *et al* 2020(11)	pJ404
pJ404FusBCTD-R241K	This work	pJ404FusBCTD
pJ404FusBCTD-R241A	This work	pJ404FusBCTD
pJ404FusBCTD-R242K	This work	pJ404FusBCTD
pJ404FusBCTD-H317A	This work	pJ404FusBCTD
pJ404FusBCTD-D322N	This work	pJ404FusBCTD
pExpFusBCTD-SpFer	This work	pExp
pJ404SpFer	This work	pJ404
pExpNoTevSpFer	This work	pExp
pExpTEVSpFer	This work	pExp
pWaldoFusANTR-GFP	Wojnowska *et al* 2020(11)	pWaldo
pWaldoFusANTR-GFP-D53A	This work	pWaldoFusANTR-GFP

Table S4. **List of primers used in this study.**

TARGET PLASMID	PRIMER NAME	PRIMER SEQUENCE (5’à3’)
pJ404FusBCTD-R241K	FusB_R241K_F	CAGAGCACTGCATAAACGCGTGAATTATC
	FusB_R241K_R	GATAATTCACGCGCTTATGCAGTGCTCTG
pJ404FusBCTD-R241A	FusB_R241A_F	CAGAGCACTGCATGCGCGCGTGAATTATC
	FusB_R241A_R	GATAATTCACGCGCGCATGCAGTGCTCTG
pJ404FusBCTD-R242K	FusB_R242K_F	GAGCACTGCATCGCAAAGTGAATTATCCCTC
	FusB_R242K_R	GAGGGATAATTCACTTTGCGATGCAGTGCTC
pJ404FusBCTD-H317A	FusB_H317A_F	CAAATTGAATGGAGCGATTCGGGTTGATGAC
	FusB_H317A_R	GTCATCAACCCGAATCGCTCCATTCAATTTG
pJ404FusBCTD-D322N	FusB_D322N_F	CATTCGGGTTGATAACCAGCAACTCGAGC
	FusB_D322N_R	GCTCGAGTTGCTGGTTATCAACCCGAATG
pExpFusBCTD-SpFer	FusionCTD_F	GAAAACCTGTACTTCCAGGCAGCTCGTGGTGCAGGGAAAAG
	FusionCTD_R	GAACCCGCGGAACCTTGCTGGTCATCAACCCGAATG
	FusionSpF_F	CAGCAAGGTTCCGCGGGTTCCGCTGG
	FusionSpF_R	GAACTGCGGGTGGCTCCATTAGGCAGTGAGCTCCTCTTC
pJ404SpFer	pJ404SpFer_F	GGAATTCCATATGGCGGCTTACAAGGTCACCC
	pJ404SpFer_R	CACCTCGAGTGCCGTCAGTTCTTCTTCTTTATG
pExpNoTevSpFer	pExpTevSpFer_F	CACCATCACCATCACCATATGGCGGCTTACAAGGTCACC
	pExpSpFer_R	GAACTGCGGGTGGCTCCATTATGCCGTCAGTTCTTCTTC
pExpTevSpFer	pExpTevSpFer_F	CGAAAACCTGTACTTCCAGGCGATGGCGGCTTACAAGGTCACCCTG
	pExpSpFer_R	GAACTGCGGGTGGCTCCATTATGCCGTCAGTTCTTCTTC
pWaldoFusANTR-GFP-D53A	FusA_D53A_F	GGAAAACGGCGAAGCGACGATTTTAGTCC
	FusA_D53A_R	GGACTAAAATCGTCGCTTCGCCGTTTTCC

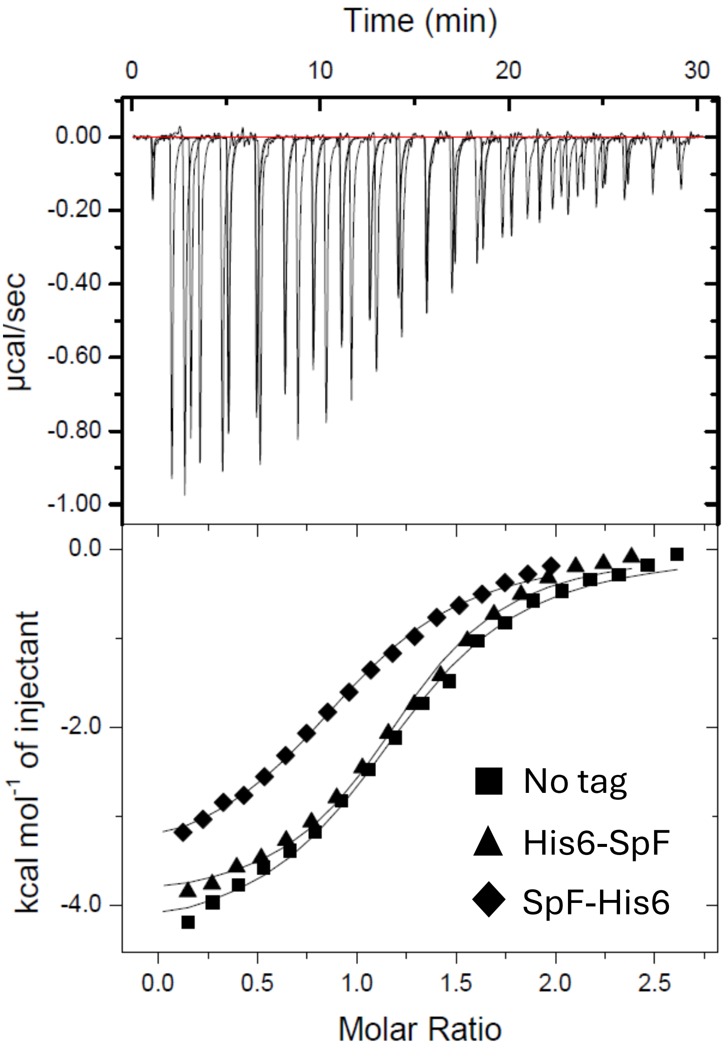

Figure S8. **Binding of different SpFer constructs to FusB-CTD.** Thermograms from the titration of tag-free (squares), N-terminally-tagged (triangles) and C-terminally tagged SpFer (diamonds, K_D = 18.1±4.6 μM) into *Pc*FusB-CTD. Titrations of tag-free and N-terminally tagged SpFer were virtually indistinguishable, with the average K_D = 7.8±0.5 μM.

Table S5. List of all strains from 13 different genera used in the conservation analysis. The number of copies as well as accession numbers of *tonB* and *fusB* are shown in the corresponding columns.

(TABLE IN A SEPARATE EXCEL FILE)

Table S6. TonB sequences used for the generation of sequence logos. Table listing 4275 TonB sequences from Genbank datasets that were used to generate the sequence logos of "canonical", "additional" and "other" TonBs (Supplementary Figure 1). The canonical TonBs were verified as being encoded as stand-alone genes and therefore likely to service multiple TBDRs. FusB sequences are contained within the additional class. The 7 residues including the conserved YP motif in each TonB are shown.

(TABLE IN A SEPARATE EXCEL FILE)

Figure S9. AlphaFold3 prediction of FusA-substrate complexes. A – PAE plots for the three predicted complexes, including AraFer with *P. atrosepticum* SCRI1043 FusA. B - AlphaFold3-predicted complex of *Pc*LMG2410 FusA with ferredoxin (red) and pectocin M1 (grey), with 90° rotation between the two views. C – HADDOCK-derived *Pa*SCRI1043 FusA-AraFer complex. The buried surface area and the number of direct polar contacts (B and C) were determined using PISA server. D - overlay of AraFer complexes with *Pc*LMG2410 (yellow/red) and *Pa*SCRI1043 FusA (blue/magenta). E – polar contacts between AraFer and FusA in the AF3 prediction. Both proteins are in grey, only part of FusA is shown.

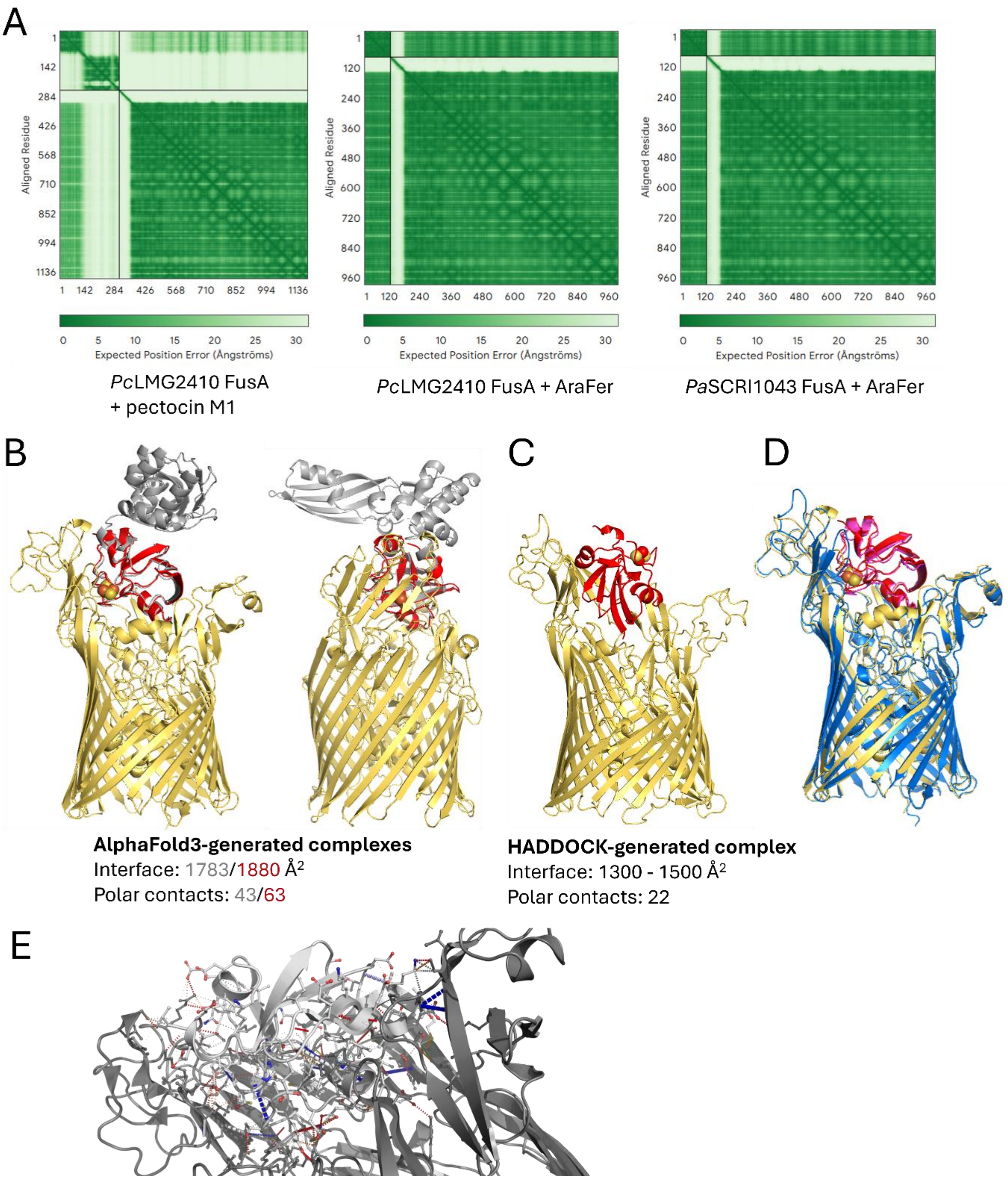

Figure S10. Raw ITC data.

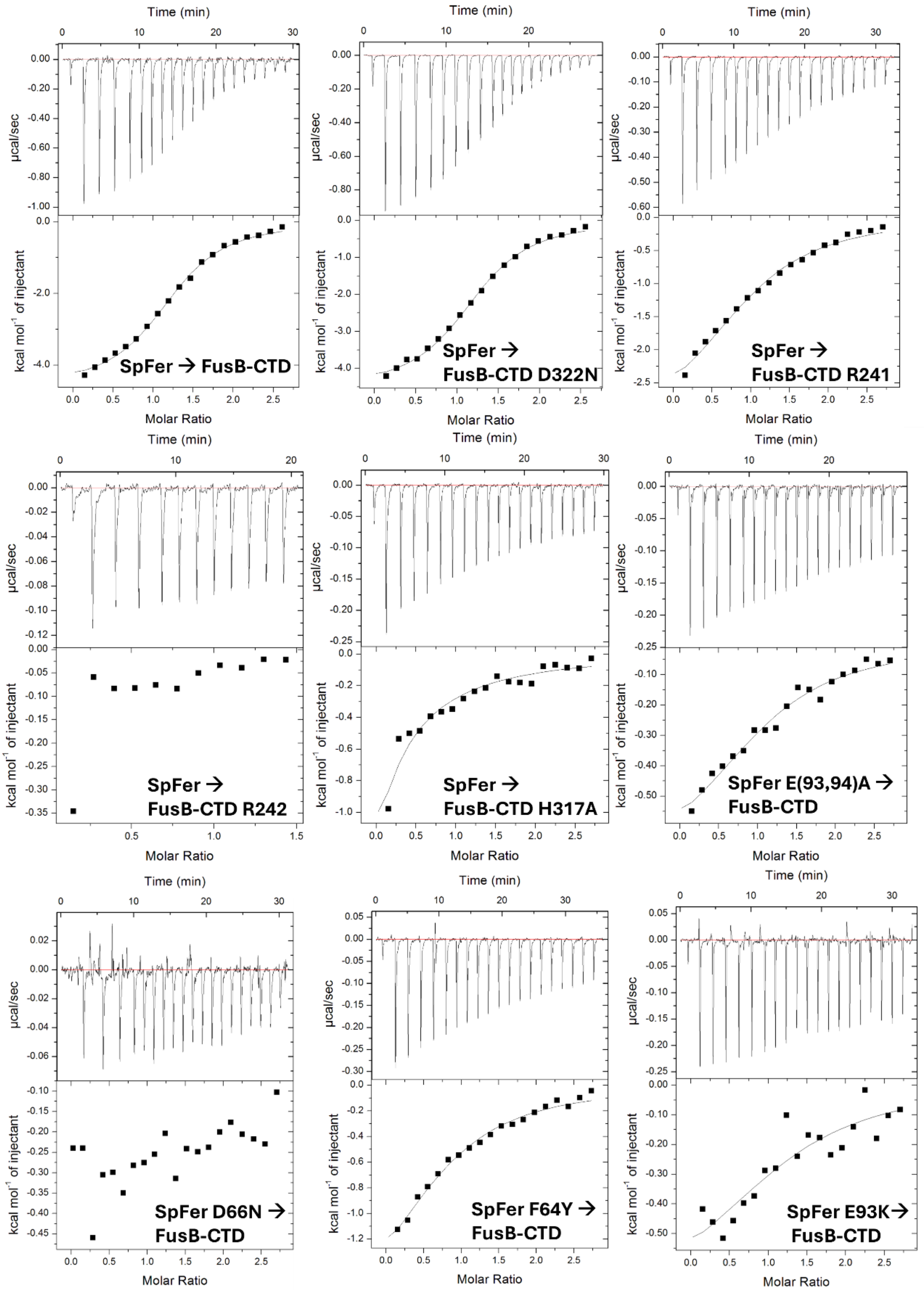

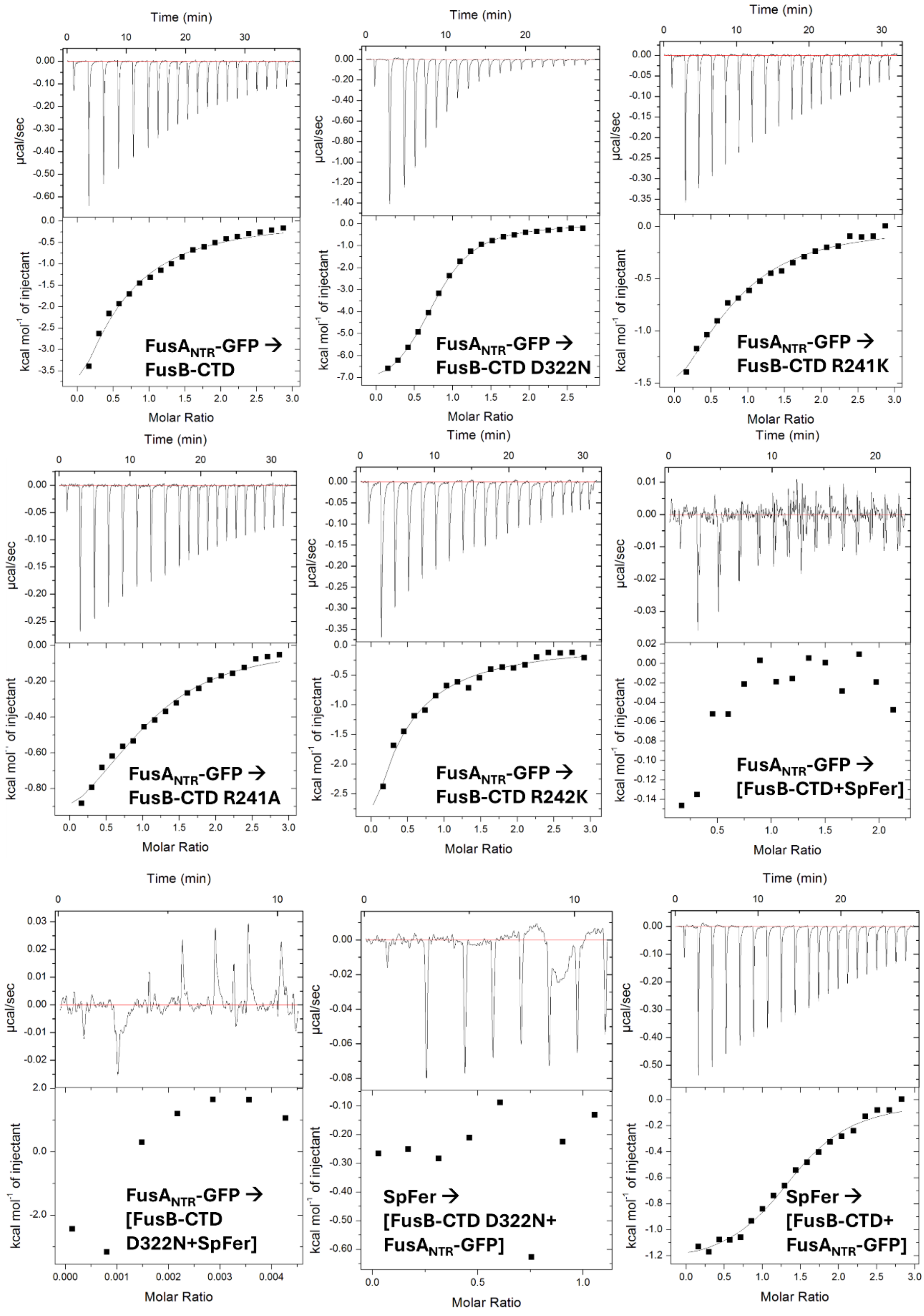
Time (min)
µcal/sec
kcal mol^{-1} of injectant
Molar Ratio
FusA$_{NTR}$-GFP → FusB-CTD
FusA$_{NTR}$-GFP → FusB-CTD D322N
FusA$_{NTR}$-GFP → FusB-CTD R241K
FusA$_{NTR}$-GFP → FusB-CTD R241A
FusA$_{NTR}$-GFP → FusB-CTD R242K
FusA$_{NTR}$-GFP → [FusB-CTD+SpFer]
FusA$_{NTR}$-GFP → [FusB-CTD D322N+SpFer]
SpFer → [FusB-CTD D322N+ FusA$_{NTR}$-GFP]
SpFer → [FusB-CTD+ FusA$_{NTR}$-GFP]

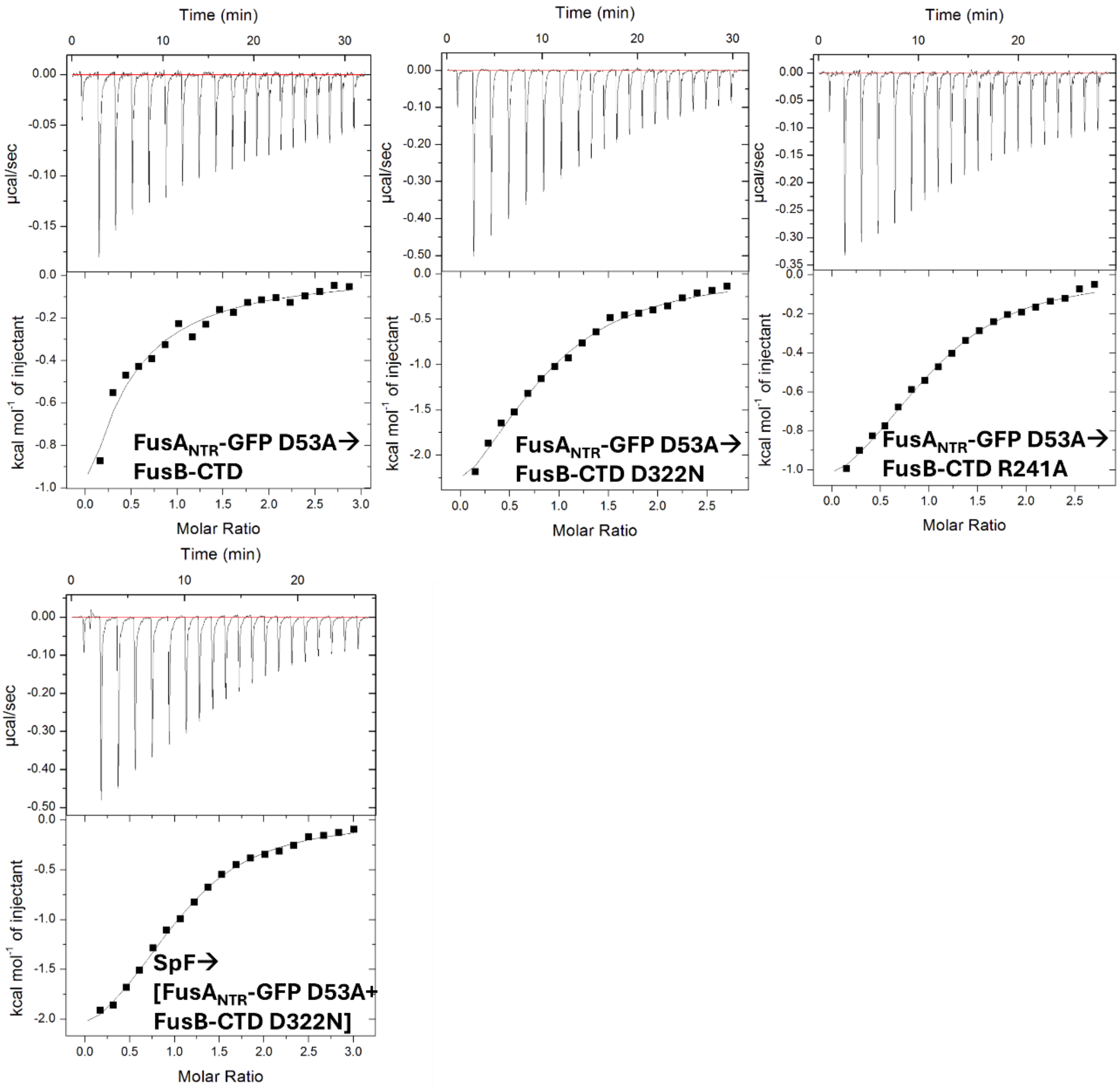
Time (min)
µcal/sec
kcal mol^{-1} of injectant
Molar Ratio
FusA$_{NTR}$-GFP D53A→
FusB-CTD
FusA$_{NTR}$-GFP D53A→
FusB-CTD D322N
FusA$_{NTR}$-GFP D53A→
FusB-CTD R241A
SpF→
[FusA$_{NTR}$-GFP D53A+
FusB-CTD D322N]
